# Response to “Bio-optimized *Curcuma longa* extract is efficient on knee osteoarthritis pain: a double-blind multicenter randomized placebo controlled three-arm study”

**DOI:** 10.1186/s13075-020-2108-3

**Published:** 2020-02-11

**Authors:** Jean-Pierre Pelletier, Jean-Pierre Raynauld

**Affiliations:** 1 Osteoarthritis Research Unit, University of Montreal Hospital Research Centre (CRCHUM), 900 Saint-Denis, Suite R11.412, Montreal, Quebec H2X 0A9 Canada; 2 Institut de Rhumatologie de Montréal, 1551 Ontario Street East, Montreal, Quebec H2L 1S6 Canada

In the last issue of *Arthritis Research & Therapy*, Henrotin et al. [1] report the outcomes of a double-blind multicenter randomized placebo controlled, three-arm study assessing the effect of two doses of bio-optimised *Curcuma longa* (BCL) on the symptoms of knee osteoarthritis (OA). They conclude in the “key messages” that BCL is an efficient and safe treatment to relieve pain in knee OA patients.

It is our perception that this conclusion is likely to be misleading, as it is not supported by the content of the article.

To begin with, this trial, which is an exploration study initiated on May 2014, has been registered retrospectively on September 21, 2016, a major abnormality which should have been but is not explained in the paper. It is not a common practice to register a clinical trial more than 2 years after the study start date, as it is well known that this practice has been implemented to be a safeguard of the overall conduct of the study.

Importantly, the authors selected two co-primary endpoints, i.e. Patient Global Assessment of Disease Activity (PGADA) and serum sColl2-1, to be assessed as the changes observed from baseline to day 90 of treatment. The choice of the co-primary endpoints selected by the authors is unusual, being different from those recommended by regulatory agencies or by most respected scientific organisations, i.e. pain and function.

In the intention-to-treat (ITT) analysis, the changes observed in both co-primary endpoints, in each of the BCL treatment groups, failed to reach the level of statistical significance compared to the changes observed in the placebo group. Therefore, in terms of efficacy, this study should be considered as a failure. Moreover, it is scientifically unsound to conduct post hoc tests or to re-analyse the primary endpoints in secondary populations (full analysis set or per-protocol population) when the primary endpoints are not met in the ITT population.

According to the method, the study was registered as a 6-month study and was conducted as such, as shown in the flow chart of the study (Fig. 1). However, the authors do not give any information on the outcomes observed between the primary analysis (month 3) and the end of study (month 6), except for a rather high, hence suspicious rate of withdrawals between month 3 and month 6, raising safety concerns about the BCL treatment.

At month 3, the authors report significantly more adverse events related to the product in the BCL high dose than in the BCL low dose and placebo, with abdominal discomfort and diarrhea being the most frequently reported adverse events. Knowing that the plateau curcumin levels were somewhat comparable in the high- and low-dose BCL groups, such results generate major concerns about the gastro-intestinal safety of the product.

Moreover, the authors originally planned a dropout rate of 15%, and they faced significantly higher dropout rates, i.e. 23.4% in the placebo group, 34.7% in the BCL low-dose group and 38.9% in the high BCL dose group. The reasons for dropouts are not properly explained in Fig. 1, e.g. *n* = 21 discontinued in the ITT BCL high-dose group but only 18 reasons for dropout provided in Fig. 1.

In summary, we have several major concerns about the design and concept of this study and about the support of the conclusions by the scientific evidence provided.

An appropriate conclusion would be that this study does not support that the actual *Curcuma longa* formulation used in this study is an effective treatment for the management of symptoms in knee osteoarthritis. Moreover, serious concerns are raised regarding the innocuity of the compound.

## Data Availability

N/A
